# Influence of collagen and some proteins on gel properties of jellyfish gelatin

**DOI:** 10.1371/journal.pone.0253254

**Published:** 2021-06-18

**Authors:** Artima Lueyot, Vilai Rungsardthong, Savitri Vatanyoopaisarn, Pokkwan Hutangura, Benjamaporn Wonganu, Pisit Wongsa-Ngasri, Sawanya Charoenlappanit, Sittiruk Roytrakul, Benjawan Thumthanaruk

**Affiliations:** 1 Department of Agro-Industrial, Food, and Environmental Technology, Faculty of Applied Science, King Mongkut’s University of Technology North Bangkok, Bangsue, Bangkok, Thailand; 2 Department of Biotechnology, Faculty of Applied Science, King Mongkut’s University of Technology North Bangkok, Bangsue, Bangkok, Thailand; 3 Fishery Technological Development Division, Department of Fisheries, Ministry of Agriculture and Cooperatives, Chatuchak, Bangkok, Thailand; 4 Functional Ingredients and Food Innovation Research Group, National Center for Genetic Engineering and Biotechnology, National Science and Technology Development Agency, Klong Luang, Pathumthani, Thailand; Brandeis University, UNITED STATES

## Abstract

Marine gelatin is one of the food proteins used in food and non-food products, offering desirable functionalities such as gelling, thickening, and binding. Jellyfish has been chosen for this gelatin research, in view of the benefits of its main collagen protein and lower fat content, which may reduce the amounts of chemicals used in the preparative steps of gelatin production. To date, the lack of identified proteins in gelatin has limited the understanding of differentiating intrinsic factors quantitatively and qualitatively affecting gel properties. No comparison has been made between marine gelatin of fish and that of jellyfish, regarding protein type and distribution differences. Therefore, the study aimed at characterizing jellyfish gelatin extracted from by-products, that are i.e., pieces that have broken off during the grading and cleaning step of salted jellyfish processing. Different pretreatment by hydrochloric acid (HCl) concentrations (0.1 and 0.2 M) and hot water extraction time (12 and 24 h) were studied as factors in jellyfish gelatin extraction. The resultant jellyfish gelatin with the highest gel strength (JFG1), as well as two commercial gelatins of fish gelatin (FG) and bovine gelatin (BG), were analyzed by liquid chromatography-tandem mass spectrometry (LC-MS/MS). The results show that the jellyfish gelatin (JFG1) extracted with 0.1 M HCl at 60°C for 12 h delivered a maximum gel strength of 323.74 g, which is lower than for FG and BG, exhibiting 640.65 and 540.06 g, respectively. The gelling and melting temperatures of JFG1 were 7.1°C and 20.5°C, displaying a cold set gel and unstable gel at room temperature, whereas the gelling and melting temperatures of FG and BG were 17.4°C, 21.3°C, and 27.5°C, 32.7°C, respectively. Proteomic analysis shows that 29 proteins, of which 10 are types of collagen proteins and 19 are non-collagen proteins, are common to all BG, FG, and JFG1, and that JFG1 is missing 3 other collagen proteins (collagen alpha-2 (XI chain), collagen alpha-2 (I chain), and collagen alpha-2 (IV chain), that are important to gel networks. Thus, the lack of these 3 collagen types influences the inferior gel properties of jellyfish gelatin.

## Introduction

Gelatin is the only protein type among hydrocolloids that functions similarly to other food carbohydrate-based hydrocolloids. The increase in the number of food, pharmaceutical, and cosmetic products has led to an increase in the use of gelatin to some 450 kilotons in 2018 for gelling, stabilizing, emulsifying, and water-binding [1–3]. At the commercial scale, the gelatin production sources have been produced from by-products of mammalian animals, including pigs and cows, by partial hydrolysis of skin, cartilage, and bones [4]. With a great concern for food safety, questions regarding bovine spongiform and foot and mouth diseases associated with bovine gelatin, as well as strict religious issues of porcine gelatin [1–5], have driven the use of by-products of fish from marine sources as an alternative commercial gelatin. Fish gelatin has also been potentially certified in accordance with kosher and halal regulations [6, 7]. In addition to fish gelatin from commercially available tilapia, marine species such as Hoki (*Macruronus novaezelandiae*) [6], clown featherback [7], unicorn leatherjacket [8, 9], Atlantic salmon [10], bigeye snapper [11–13], brown stripe red snapper [13], seabass [14], catfish [15], tuna [5, 16], brown-banded bamboo shark [17], and blacktip shark [17], and jellyfish [18–21] have been researched as other sources of commercial gelatin production. However, such marine gelatins have shown inferior gel strength and thermal stability (gelling and melting temperatures) compared to bovine and porcine gelatins. Thus, improved preparation processes and sophisticated biochemical analysis are still needed to produce better gelatin quality.

Gel properties, including gel strength, viscosity, and thermal stability, are essential food texture and structure attributes. Gelatin gel is a thermo-reversible gel where the denatured coils of collagen form a network structure maintained by hydrogen bonds within junction zones [22]. Reviews of factors affecting gelatin quality are the source of marine raw material; chemicals used for pretreatment; pH; and extraction conditions of temperature and time [1–5]. Among one of the marine animals, jellyfish has been researched concerning gelatin. Jellyfish is a human food [23], and jellyfish fisheries have been reported worldwide with an estimated production value of US$ 100 million [24]. Of Asian countries, Thailand is the leading country in processing jellyfish for food [19, 20]. With the benefit of minimal fat content, the preparation step for jellyfish gelatin production does not require excessive chemical treatment [18–20]. However, jellyfish gelatin’s gel strength is from 47–118 g [18–21], while most fish gelatin samples were reported as having gel strength higher than 200 g, including gelatins from brown stripe red snapper, catfish, brown-banded bamboo shark, and blacktip shark [13, 15, 17]. To date, the predictability of weak gel behavior of jellyfish gelatin, based on the identification of proteins by the mass spectrometric method, is possible but not well reported. Detecting and identifying the authenticity of porcine and bovine gelatin in yogurt, cheese, and ice cream were performed by NanoUPLC-ESI-Q-TOF-MS [25]. The proteomic results deliver both quantitative and qualitative information. The drawback of this technique is that it requires sophisticated equipment and software needed for interpreting the data, and is thereby considered an expensive analysis method. However, the identification of gelatin proteins affecting gel properties is necessary to integrate the chromatographic method and mass spectroscopy method, to understand how different gelatin sources exhibit different gel properties. No experiments have demonstrated and compared jellyfish gelatin proteins with other commercial gelatins by a proteomic approach.

Therefore, the objectives of this study were to characterize jellyfish gelatin produced from by-products and to compare the proteins in jellyfish (JFG) and commercial fish gelatin (FG) and bovine gelatin (BG) by liquid chromatography-tandem mass spectrometry (LC-MS/MS). The proteomic analysis data could be a method of choice to differentiate gelatin proteins from each raw material source exhibiting different gel properties.

## Materials and methods

### Sample preparation

50 kg of dried minced jellyfish (*Lobonema smithii*) by-products from salted jellyfish processing were obtained from Mahachai Seafood Co., Ltd, Thailand. This raw material of by-products was subjected to multiple tap water washes to reduce salt and other impurities. The cleaned samples were then drained and dried at 60°C using a tray dryer (Dwyer, TDII, Thailand) for 24 h. The dried sample was ground and passed through a sieve of 35 mesh. The resulting jellyfish powder was kept in a polyethylene bag at room temperature before use [26]. The method of gelatin production was modified from the previous studies [19, 20]. The jellyfish powder was agitated in 0.05 M NaOH solution in the ratio of 1:15 (w/v) using a controlled refrigerated shaker (WiseCube, WIS-20R, Korea) at 4°C for 2 h with an agitation speed of 150 rpm. After several washes, the washed samples with a neutral pH were agitated in two different HCl concentrations of 0.1 and 0.2 M in the ratio of 1:10 (w/v) for 2 h at room temperature. The samples were rewashed and subjected to hot water extraction of 60°C at a ratio of 1:10 (w/v) for 12 and 24 h in a shaker water bath (Memmert, Schwabach, Germany) with a speed of 70 rpm. The extracted jellyfish gelatin solutions were filtered, and the filtrates were dried at 60°C using a tray dryer (Dwyer, TDII, Thailand). The dried jellyfish gelatin sheets, with a moisture content of 6.8%, were packed in a polyethylene bag and stored in a desiccator for further experiment. The commercial fish gelatin and bovine gelatin were food-grade; they were obtained from Nutrition Sc. Co., Ltd (Thailand).

### Analysis of jellyfish gelatin gel properties

#### Gel strength

The 6.67% gelatin gel was prepared and transferred to a setting mold having dimensions of 3 cm in diameter and 2.5 cm in height, according to the GMIA method [27]. The gel was incubated at refrigerated temperature (4°C) for 18 h before measuring gel strength using the texture analyzer (Stable Micro System, Surrey, UK). The measurement was performed as previously described [19].

#### Moisture content

The moisture content of the jellyfish gelatin powders was determined according to the method of AOAC [28]. Jellyfish gelatin (3 g) was dried in an oven at 105°C until reaching a steady weight, and the moisture content was calculated by weight difference before and after the drying process.

#### Viscosity

Gelatin solutions (6.67% w/v) were prepared in distilled water following heating to 60°C. The viscosity (cP) of 10 ml of gelatin solutions was evaluated using a Brookfield viscometer (DV-II, Brookfield Scientific Promotion Co., Ltd. Middleboro, MA) operated at 90 rpm and 25°C [29].

#### Thermal stability

The thermal stability of the gelatin gel was analyzed for the gelling and melting temperatures, prepared at 6.67% solution. A parallel plate (40 mm) with a gap size of 0.5 mm was set up in oscillatory mode for the deformation oscillatory measurement using a controlled stress rheometer (Gemini 200 HR Nano, Malvern Instruments, UK). The temperature was varied from 40°C to 5°C. When the temperature reached 5°C, it was held for 10 min and then increased further to 40°C at a rate of 2°C per min. The resulting gelatin solution was poured into the parallel plate and covered with paraffin oil [30]. The data of elastic modulus (G’), loss modulus (G”) and phase angle (δ) were generated during cooling and heating. The gelling and melting temperatures were calculated where tan δ became 1 [30].

#### Gelatin clarity

The gelatin solution was prepared with some modification by solubilizing the 6.67% (w/v) concentration of gelatin solution in distilled water at 60°C [31]. The turbidity of the gelatin solution was read at 620 nm using a spectrophotometer (SP– 820, Metertek, Taipei, Taiwan).

#### Color

Color measurement of the jellyfish gelatin solution prepared at 6.67% (w/v) with the highest gel strength was analyzed using a Hunter Lab (Color Quest XE, USA), displaying values of L*, a*, b*, indicating lightness/brightness (0 = black, 100 = white), redness (+a) /greenness (-a), and yellowness (+b) /blueness (-b). The color value of L*, a*, and b* were expressed in chroma, with hue angle (H^o^) according to formulas (1) and (2).


$$Chroma=\surd (a^{*}+b^{*})$$ (1)

$$H^{o}=tan^{‐1}(b^{*}/a^{*})$$ (2)

#### Determination of isoelectric point (IEP)

The isoelectric point (IEP) of gelatin was determined according to the flocculation observation method with slight modification, as previously described [32]. A 4% gelatin solution was prepared at 60°C. Each solution (5 ml) of 0.1 M acetate buffer was prepared at pH 3 to pH 10, and the hot gelatin solution (2 ml) was added to a test tube and mixed well. The mixture was allowed to cool down and then titrated with 99.5% ethanol until faint white turbidity persisted. The pH value and ethanol volume used at the first flocculation were taken. The IEP of the gelatin sample was recorded.

#### Fourier transform infrared (FTIR) spectroscopic analysis

The jellyfish gelatin sample with the highest gel strength was selected for determining changes in functional groups using an FTIR spectrometer (Jasco Inc., Easton, MD, USA). The sample was prepared by mixing jellyfish gelatin powder with KBr to form pellets using an MP-1 hydraulic press (JASCO Corporation, Tokyo, Japan). The scanning spectra were in the range of 4000 and 400 cm^-1^.

#### Protein identification by LC-MS/MS

Dried gelatin samples from jellyfish having the highest gel strength, as well as fish and bovine samples, were desalted by dialysis overnight. Then, each gelatin sample was digested with sequence grade trypsin (ratio of 1:20) (Promega, Germany) at 37°C overnight, dried, and dissolved with 0.1% formic acid. The protein concentration of all gelatin samples was measured using the Lowry assay [33]. For LC-MS/MS analysis, each of the tryptic peptide samples (100 ng) were injected in triplicate into an UltimateTM 3000 Nano/Capillary LC System (Thermo Scientific) coupled to a Hybrid quadrupole Q-TOF impact II™ (Bruker Daltonics, Germany) equipped with a Nano-captive spray ionization (CSI) source. The peptides were enriched on a μ-Precolumn PepMap100 5 μm, 0.3 μm i.d. × 5 mm, pore size 100 ^o^A and separated using a PepMap^®^ RSLC C18, 3 μm, 75μm i.d. × 150 mm, pore size 100 ^o^A nanoViper (Thermo Scientific, USA). Two mobile phases were: A, 0.05%TFA in water and B, 0.05%TFA in 80% acetonitrile for chromatography. A linear gradient of 5–55% solvent B was run for over 45 min at a flow rate of 300 μl/min, and a column temperature of 40°C was used to elute the peptides.

For mass identification, electrospray ionization was performed at 1.4 kV using the Captive Spray Mass spectra (MS), and MS/MS spectra were achieved in the positive-ion mode over the range (m/z) 150–2,200 (Compass 1.9 software, Bruker Daltonics). MaxQuant (version 1.6.6.0) was used to quantify individual samples bioinformatically, and their MS/MS spectra were matched to the UniProt database using the Andromeda search engine [34]. The protein sequences assigned with protein IDs with known/putative functions from the UniProt database were denoted as annotated proteins. Label-free quantitation with MaxQuant settings was performed, which included (1) a maximum of two missed cleavages, (2) mass tolerance of 0.6 Daltons for the main search, (3) trypsin as the digestive enzyme, (4) carbamidomethylation of cysteine residues as a fixed modification, and (5) oxidation of methionine and acetylation of the protein N-terminus as variable modifications. Notably, peptides with a minimum of 7 amino acids and at least one unique peptide were required for protein identification. The protein false discovery rate (FDR) was set at 1% and estimated from the reverse searches of sequences. The maximal number of modifications per peptide was set to 5. For searching in FASTA files, a protein database of fish gelatin, bovine gelatin, and bovine collagen, alpha 2 and 1 chain were downloaded from UniProt. A database with potential contaminants included in MaxQuant was automatically added. The MaxQuant ProteinGroups.txt file was loaded into Perseus version 1.6.6.0 [35], and potential contaminants that did not correspond to any UPS1 protein were removed from the data set. Max intensities were log2 transformed, and pairwise comparisons between conditions were made via t-tests. Missing values were also imputed in Perseus using constant value (zero). The visualization and statistical analyses were conducted using a MultiExperiment Viewer (MeV) in the TM4 suite software [36]. The Venn diagram displays the differences between protein lists originating from different samples [36].

#### Determination of soluble protein concentration

The soluble gelatin was measured using the Lowry assay [33] with bovine serum albumin as a standard.

### Statistical analysis

The analysis of variance (ANOVA) and differences of mean values calculated using Duncan’s multiple range test were analyzed using SPSS software (SPSS 17.0 for windows, SPSS Inc., Chicago, IL, USA).

## Results and discussion

### Physicochemical properties of jellyfish, bovine, and fish gelatins

#### Gel strength

Table 1 shows the physicochemical properties of JFG1, FG, and BG. Results for all three gelatins varied in all determinations. In this study, gel property determination focuses on gel strength, viscosity, and thermal stability. The quality of jellyfish gelatin extracted from by-products of salted jellyfish (JFG) was compared to commercial products of fish gelatin (FG) and bovine gelatin (BG). The factors studied that affected jellyfish gelatin were varied HCl concentration (0.1 and 0.2 M) and extraction time (12 and 24 h), labeled as JFG1, JFG2, JFG3, and JFG4, under control extraction temperature of 60°C. Results show that all jellyfish gelatin samples appeared in a soft gel. The JFG1 sample with a short extraction time of 12 h yielded the highest gel strength of 323.74±7.02 g, greater than any other extracted gelatin samples. The JFG2 sample was extracted for 24 h resulting in a gel strength of 236.85±9.72 g. The JFG4 sample treated at a higher concentration of HCl (0.2 M) yielded a higher gel strength of 182.83±4.60 g compared with the JFG3 treated with 0.1 M HCl having a gel strength of 85.32±5.00 g. Following the production process of gelatin, the resulting gel strength can be affected by several critical factors, including the extraction procedure, type of raw materials used, differences in the proportion of hydroxyproline and proline content in each raw material, the molecular weight distribution of peptide, and the chain length of denatured collagen [2–4, 8, 16]. Unlike other initial raw materials, jellyfish have been studied for collagen and gelatin research, in which their bodies, apart from water, have high collagen and less fat content. Collagen is an initial raw material protein for producing gelatin by a chemical and thermal process. High temperature causes hydrolysis and destroys the bonding of the triple helix of the collagen bundle. Long extraction time denatures collagen and generates short gelatin peptides, thereby effectively preventing a gel network from forming [12].

**Table 1:** Physicochemical properties of jellyfish gelatin (JFG1), fish gelatin (FG), and bovine gelatin (BG).

Property	JFG1*	FG	BG
Gel strength (g)	323.74 ± 2.041^c^	640.65 ± 2.18^a^	540.06 ± 3.69^b^
Viscosity (cP)	7.73 ± 0.06^c^	15.67 ± 0.12^b^	28.73 ± 0.12^a^
Gelling temperature (^o^C)	7.1	17.4	21.3
Melting temperature (^o^C)	20.5	27.5	32.7
Isoelectric point (pH)	7.0	6.0	5.0
Clarity	0.286 ± 0.005^a^	0.107 ± 0.002^c^	0.189 ± 0.002^b^
L*	54.47 ± 0.83^c^	94.28 ± 1.73^a^	68.80 ± 0.95^b^
a*	5.88 ± 0.13^a^	0.53 ± 0.01^b^	5.96 ± 0.15^a^
b*	21.51 ± 1.19^b^	8.57 ± 0.17^c^	29.15 ± 0.71^a^
Chroma	5.23 ± 0.13^a^	3.01 ± 0.03^c^	5.92 ± 0.95^b^
Hue	74.65 ± 0.50^c^	86.44 ± 0.04^a^	78.42 ± 0.55^b^

JFG1* was from the extraction of 0.1 M HCl, 60°C for 12 h

Values are mean ± SD from triplicate determinations. Means with different letters in the same row are significantly different (p<0.05).

Regarding jellyfish, different species of jellyfish and analytical methods used resulted in different collagen content. The total collagen content of three edible jellyfish species (*Acromitus hardenbergi*, *Rhopilema hispidum*, *Rhopilema esculentum*) was in the range of 122.64–693.92 g/100 D.W. [37]. The desalted jellyfish (*Lobonema smithii*) showed 24.58% and 33.38% from the umbrella and oral arm part, respectively quantified by a microscopic measurement of hydroxyproline content and calculated on a wet weight basis [38]. The amino acid content of hydroxyproline and proline of the umbrella of desalted jellyfish (*Lobonema smithii*) was 12.85 and 17.79 mg/100 g. The content of oral arm was 16.57 and 23.81 mg/100 g crude collagen [38]. When producing gelatin, hydroxyproline and proline content were changed as a factor of acid pretreatment and extraction. Jellyfish gelatin extracted by HCl pH 1 at 45°C for 12 h showed hydroxyproline and proline of 5.62 and 4.19 g/100g [19], while that same gelatin extracted by H_2_SO_4_ pH 2 at 75°C for 12 h resulted in those amino acids at 4.63 and 3.83 g/100g [20]. Another result of gelatin extracted from *Rhopilema hispidum* showed hydroxyproline and proline of 139.3 and 81.5 residues/1,000 residues [18]. However, this study did not measure hydroxyproline and proline content of JFG1.

Regarding the species differences, the results were in agreement with those reported, that marine gelatin had lower gel strength than mammalian gelatin [5]. Most aquatic gelatins reported have gel strength in the range of 100–300 g [2]. However, in the present findings, the values of gel strength of JFG1 and the commercial fish and bovine gelatins were higher than as reported in other publications [2–4, 18–20]. The reason for this may be the 6.67% gelatin solution prepared for determination was calculated based on moisture content of 6.8%. The level of dry solid content present at a specific moisture content level may influence the strength of gelatin gel. However, most of researchers performed the analysis without concern for the moisture content of the sample. Thus, the gel strength values obtained from other reports are quite difficult to compare. Despite producing low moisture gelatin, the dried sample of JFP1 with 6.8% moisture content might be too low from a commercial perspective, resulting in expensive gelatin.

#### Viscosity

Viscosity is also an important aspect related to gel formation. Results show that JFG1 (7.73 cP) had the lowest viscosity, followed by FG (15.67 cP) and BG (28.73 cP). The viscosity values of BG and FG were about 4 and 2 times greater than that of JFG1. In this determination, the prepared sample of 6.67% was also calculated based on moisture content of 6.8%, resulting in higher viscosity values of both commercial gelatins of FG and BG. The commercial viscosity of BG reported was 9.8 cP [2]. The viscosity of commercial gelatin was acceptable at 2.0–13.0 cP [2,16], except for the viscosity of farmed giant catfish at 112.5 cP [39]. In this study, different raw material sources and different gelatin preparation may directly influence the number and molecular weight of hydrolyzed peptides [2, 4]. Also, the decrease in viscosity is affected by high temperature [18]. However, the sample preparation for analysis [16] and the test instrument [2] must also be considered for accuracy and reproducibility. Despite the low viscosity, the viscosity of jellyfish gelatin (JFG1) is in the acceptable range compared to that of other gelatins.

#### Thermal stability

**Fig 1:**
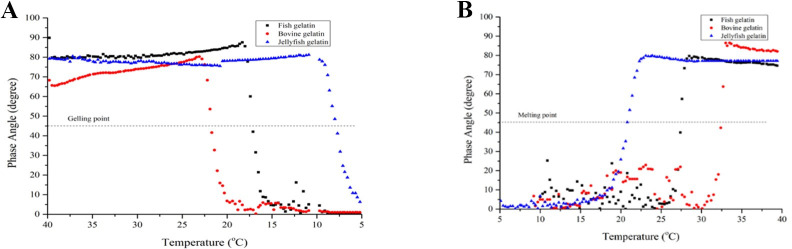
Dynamic viscoelastic profile changes in three gelatins during gelling and melting; (A) during gelling; (B) during melting.

Gelatin is a thermoreversible gel for which the melting temperature offers the unique quality of melt-in-mouth gel, and the gelling temperature is typically crucial for forming the gel network. The melting and gelling temperatures of gelatin gel are generally measured by temperature sweep tests. The temperature changes are from high (40°C) to low temperatures (5°C) and then run from low to high by a rheometer [2]. The data of elastic modulus (G’), loss modulus (G”), and degree of phase angle are calculated. Fig 1 shows the dynamic viscoelastic profile of all gelatin samples during cooling (form 40–5°C) and melting (from 5–40°C). Results show differences in gelling (Fig 1A) and melting temperatures (Fig 1B) of JFG1, JG, and BG. The JFG1 displayed the lowest gelling temperature, at 7.1°C, and the lowest melting temperature, at 20.5°C, compared to the other two gelatin gels of FG and BG (Table 1). With a specific type of jellyfish samples, the gelling temperature of the *Lobonema smithii* gelatin was much lower than that temperature of gelatin extracted from *Rhopilema hispidum* with a gelling temperature of 18°C, while the melting temperature was not much different [18]. However, in most cases, melting temperatures for mammalian gelatin were 28–31°C higher than those of aquatic gelatin reported, in the range of 16–28.9°C [2].

The temperature of gelling and melting points of gelatin gel is a crucial characteristic for producing shelf-stable food products. The low gelling and melting characteristics of jellyfish gelatin gel could be associated with lower imino and amino acid in each peptide chain, the peptide chain’s length, and the peptide’s molecular weight [3, 4]. In this finding, the soft and cold temperature set gel of jellyfish gelatin could offer possible use in refrigerated or frozen food products, similar to commercial fish gelatin. Apart from application in food products, jellyfish gelatin is applied in film formation. Extruded copolymer film composed of 3% jellyfish gelatin and 5% cassava starch solution showed that elongation of glycerol plasticized film increased greater than that of sorbitol plasticized film [21].

#### Clarity and color

**Fig 2:**
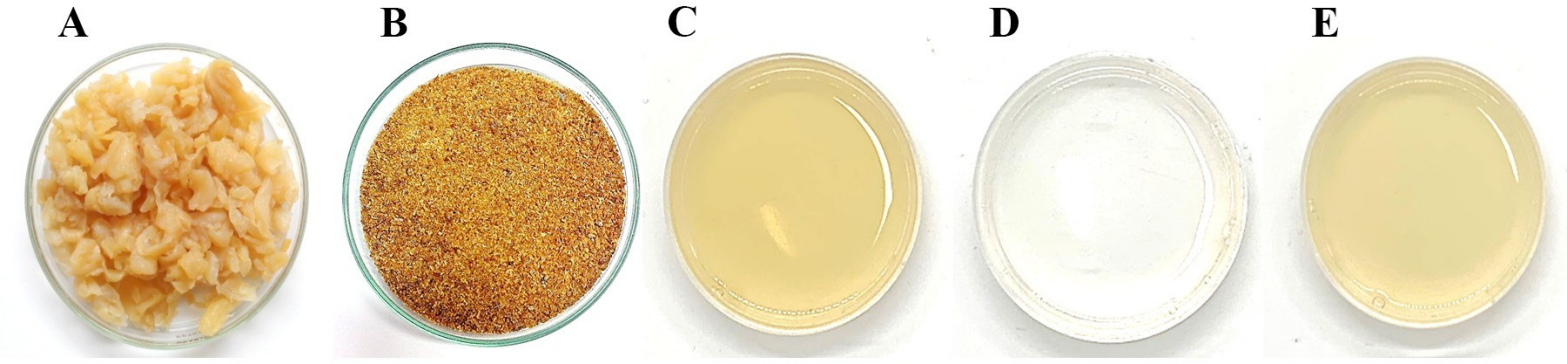
Color of jellyfish samples. (A) desalted jellyfish; (B) dried desalted jellyfish; (C) jellyfish gelatin gel; (D) fish gelatin gel, and (E) bovine gelatin gel.

The color of gelatin gel affects food color quality, and changes in its color depend on the reaction and duration time of the resultants. Fig 2A and 2B shows raw material salted jellyfish and dried desalted jellyfish of dark brown color. Indeed, a freshly caught jellyfish body exhibits translucent gel. The salt preservation changes the flaky gel umbrella and oral arms to a tender and elastic texture. By-products of salted jellyfish were produced by a processing for export. The fresh jellyfish were cured with salt, alum, and sodium bicarbonate for removing massive water in the jellyfish and dehydrating, precipitating protein and firming the texture, deodorizing, and improving a crispy texture (23,24). After a long dehydration process, the semi-dried jellyfish and by-products had a slightly brown color. For ease of use, jellyfish by-products were kept dried, thereby yielding an intense brown color.

Fig 2C–2E shows the appearance of gelatin gel of JFG1, FG, and BG. JFG1 appeared with the highest clarity gel, but not FG and BG. By measuring with a colorimeter, the JFG1 showed the lowest values of L* (lightness) and Hue compared to the values of BG and FG. The appearance of JFG1 and BG showed brown color but slight differences in b* (yellowness) (Table 1). Compared to all gelatin gel appearances, the clearest transparent gel was from FG, showing the highest L* value and the lowest a* and b* values. The dried sample used was of an intense brown color, resulting in a similar gel color. This increased color of the resulting jellyfish might be developed by the Maillard reaction, a non-enzymatic browning reaction of reducing sugar and amino acid, and it can develop as a result of high temperature and long duration time [40]. Bleaching can be applied to increase the gel’s transparency but decreases the gelling property. Squid gelatin bleached with 2% hydrogen peroxide resulted in the highest L* value and lowest gel strength [41]. In this study, no bleaching step was applied during extraction. The color was similar to the sample of type A jellyfish gelatin that appeared in yellow shades that were reported earlier [21].

#### Isoelectric point (IEP)

The IEP is where the net charge of the protein is zero. The IEP of gelatin is dependent on the process used for gelatin production and the raw material. Gelatin type A is produced by acid pretreatment, whereas gelatin type B is produced by alkaline treatment. The degree of hydrolysis of the amide group of amino acids is by acid or alkaline pretreatment, resulting in pH changes. In this study, results show JFG1 displayed at neutral pH, while the IEP values of FG and BG showed at slightly acidic pHs. The values of IEP of gelatin type A and type B varied from pH 6.5–9 and pH 4–5 [3, 41]. For commercial use, the gelatins are sold at pH 5.2–5.5 [41].

#### Fourier transform infrared (FTIR) spectra

**Fig 3:**
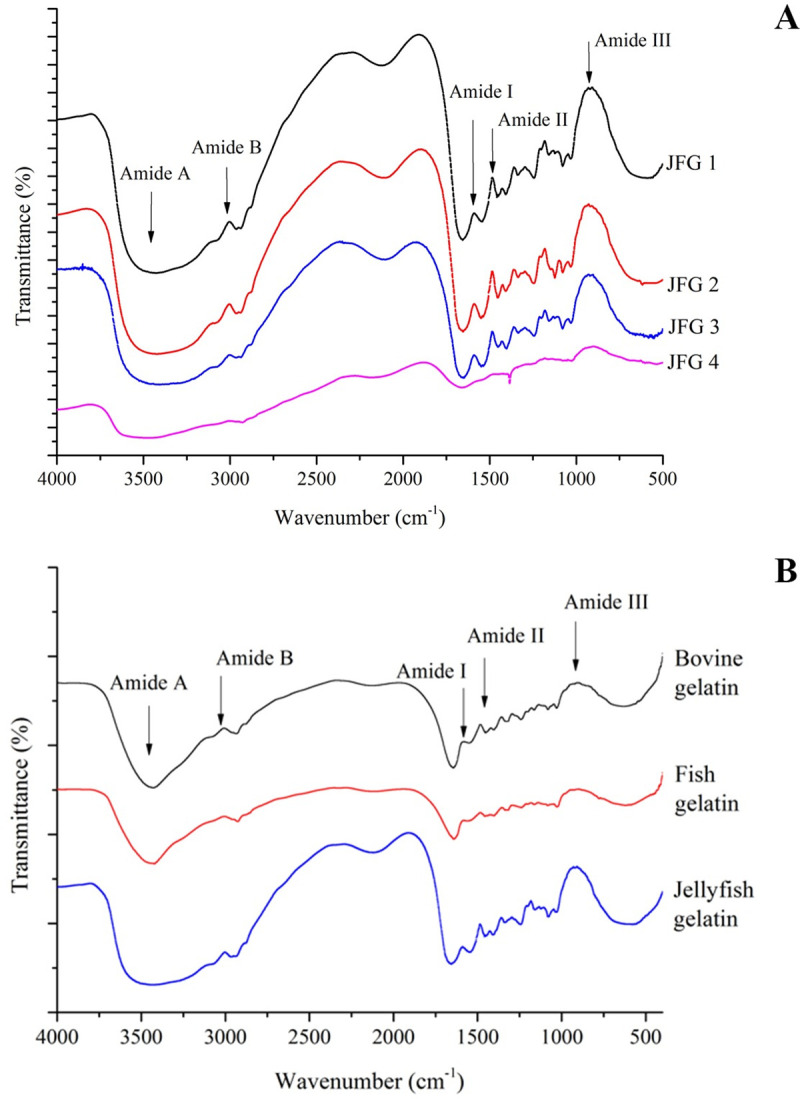
Fourier transform infrared spectra of gelatin. (A) JFG1-JFG4; with different hydrochloric acid and duration times conditions, (B) jellyfish gelatin (JFG1), bovine gelatin and fish gelatin.

The secondary structure changes at the amide region of gelatin analyzed by Fourier transform infrared (FTIR) spectroscopy are shown in Fig 3A and 3B. The characteristic transmission pattern of amide I, amide II, amide III, amide A, and amide B at the wavenumbers of 1651–1662, 1540–1560, 1230–1242, 3400–3440, and 2939 cm^-1^ was monitored. FTIR pattern spectra of extracted jellyfish gelatins (JFG1-JFG4) changed due to the concentration of HCl pretreatment and duration time of extraction (Fig 3A). The amide I and amide II vibrations are the most analyzed for protein secondary structure prediction [42]. The amide I vibration is related to a C = O stretching, and the absorption peak at amide I indicate changes in the coil structure of the gelatin [8]. The low amplitude of frequency is related to the interaction of C = O with adjacent protein chains by a hydrogen bond. The amide II vibration is related to the C-N stretch and N-H bending of the peptide bond [16]. Amide III refers to the peaks between C-N stretching and N-H deformation from amide linkages. The absorption comes from the CH_2_ group of glycine and proline. The high frequency of amplitude could be due to the change of molecular structure from the α-helical structure to random coil, showing denaturation of collagen to gelatin [42]. Amide A is related to the stretching vibration range of 3400–3440 cm^-1^ of the N H group. Amide B is involved in asymmetric stretch vibration of = C-H and -NH^3+^. In the present work, JFG1 sample displayed high gel strength (323.74 g) and showed a higher wavenumber at the amide III peak. Given the FITR results, the vigorous acid pretreatment and longer extraction time (24 h) might disrupt hydrogen bonds in the gelatin structure, causing noticeable changes in the secondary structure of the resulting gelatin. The denatured triple helix and disrupted cross-linking might occur in the telopeptide region [42].

#### Identification of proteins in jellyfish, fish, and bovine gelatin

**Fig 4:**
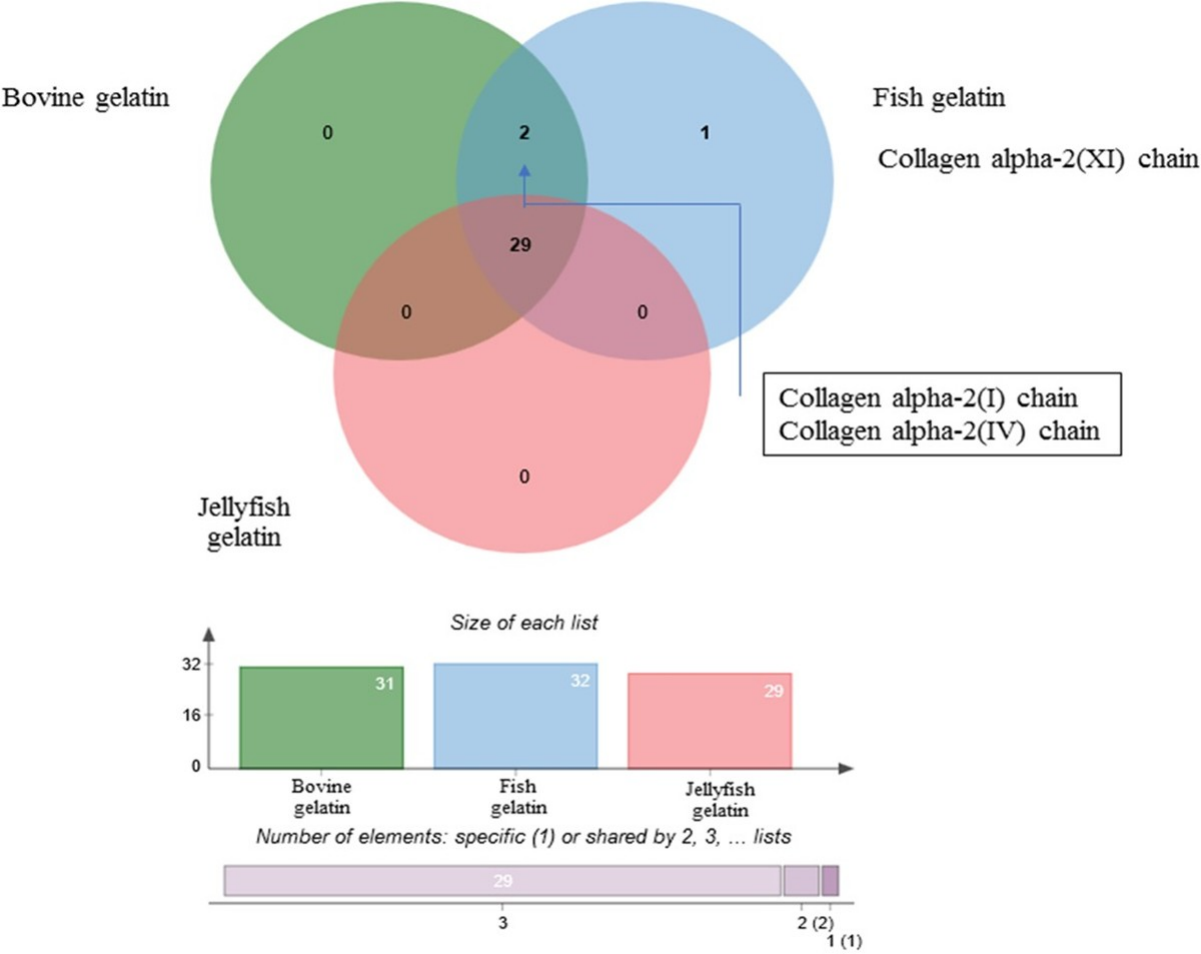
Proteins in jellyfish, fish and bovine gelatin identified by LC-MS/MS. A Venn diagram showing the total number of proteins identified in each gelatin sample.

**Fig 5:**
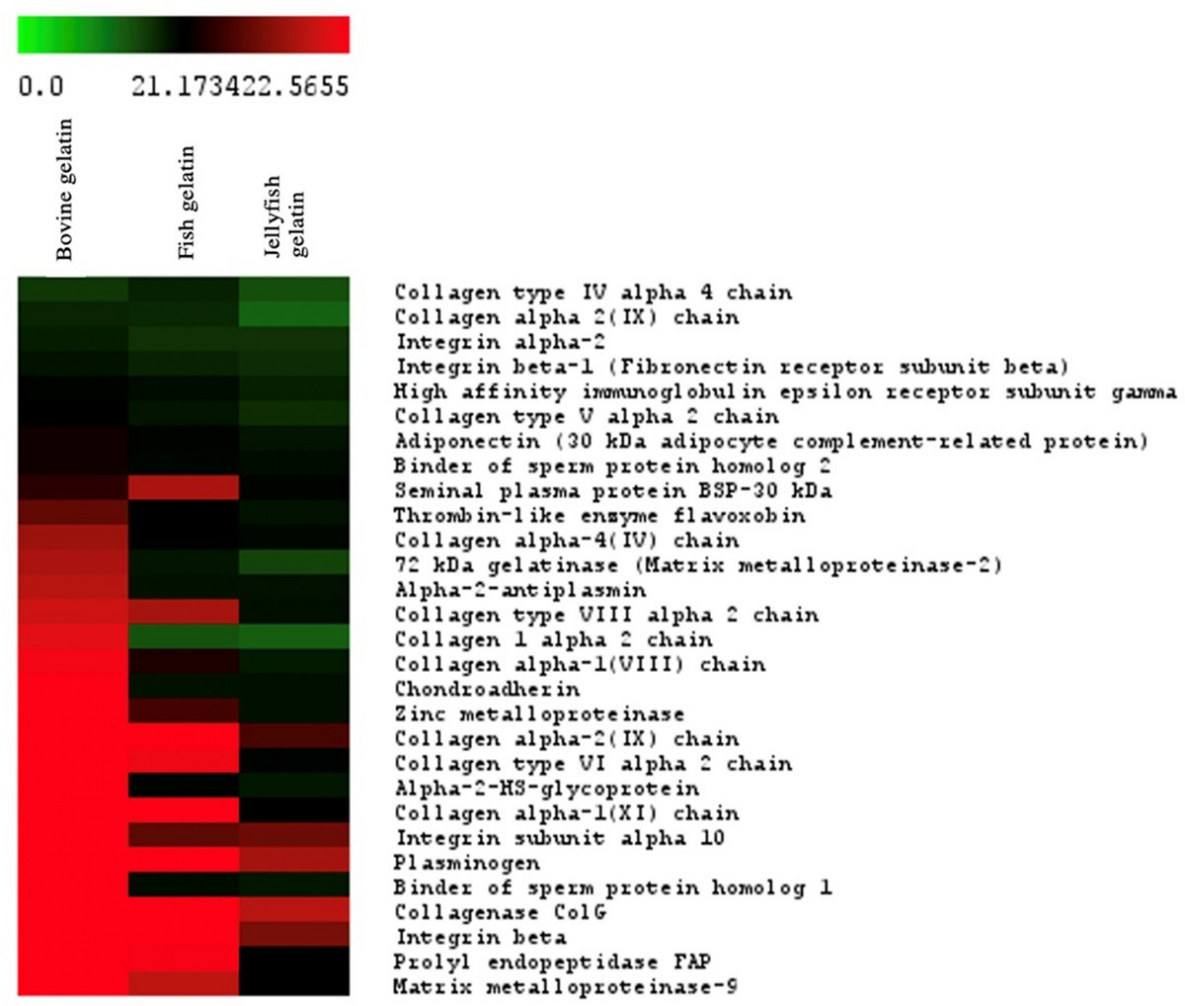
Heatmap showing the relative amount of proteins in each gelatin sample. A color bar of green to red color stands for the intensity of the total ion chromatogram from 0.0 to 22.5655.

To confirm the effects of proteins on gel strength, viscosity, gelling, and melting temperature of gelatin, a comparative study of the changes in protein patterns of JFG (originally from JFG1), FG, and BG was conducted using LC-MS/MS. A total of 32 differentially expressed proteins in JFG, FG and BG were identified. The number of unique proteins detected in each sample is shown in a Venn diagram (Fig 4). It indicates that exclusively collagen alpha-2 (XI chain) was detected in FG, while collagen alpha-2 (I chain) and collagen alpha-2 (IV chain) were present in both FG and BG. The 29 proteins commonly found in all gelatin samples with a difference in their abundance were further analyzed by Heatmap (Fig 5). The overview intensity of protein patterns in JFP was clearly different from those in FG and BG. The majority of the commonly expressed proteins, including thrombin-like enzyme flavoxobin, Collagen alpha-4 (IV) chain, 72-kDa gelatinase, Alpha-2 antiplasmin, Collagen type VIII alpha 2 chain, Collagen alpha-1 alpha 2 chain, Collagen alpha-1 (VIII) chain, Chondroadherin, Zinc metalloproteinase, Collagen alpha-2 (IX) chain, Collagen type VIalpha-2 chain, Alpha-2-MS-glycoprotein, Collagen alpha-1 (XI) chain, Integrin subunit alpha 10, Plasminogen, Binder of sperm protein homolog 1, Collagenase Col G, Integrin beta, Prolyl endopeptidase FAP and Matrix metalloproteinase-9, were present in the BG. Relatively low levels of 7 proteins, including collagen type IV alpha 4 chain, collagen alpha 2 (IX chain), Integrin alpha-2, Integrin beta-1, 72kDa gelatinase, collagen type V alpha 2 chain, and collagen 1 alpha 2 chain, were found in JFP. Higher levels of collagen alpha-2 (IX chain), Integrin subunit alpha 10, plasminogen, Collagenase Col G, and Integrin beta in jellyfish gelatin were observed in JFP; however, their relative abundance was lower than those in FG and BG. Two types of collagen alpha-2(I) chain and collagen alpha-2(IV) chain found in bovine and fish gelatin had the sequences of 20 amino acids of AGPPGPPRGAGAPGQSFLLR and 31 amino acids of AEQGEFYLLSYGSWKLNMGVPCMPEQDTQSK, respectively S1 Table.

As a result of proteomic analysis, the inferior functional properties of gel strength and gelling and melting temperatures of JFG might be due to differences in collagen type and other proteins. The intrinsic factors that may affect gel strength and melting points were amino acid composition, the alpha and beta chain ratio in the gelatin, and the gelatin peptide amount of the alpha chain [1–4]. Based on the collagen and gelatin protein pattern analyzed by SDS-PAGE, two jellyfish species (*Rhopilema hispium* and *Lobonema smithii*) were found to contain collagen type I and collagen type II [38]. However, the limitations of this analysis were an unclear protein pattern and a lack of a collagen standard.

This analysis reported for the first time that differences in collagens and other proteins directly influence gel properties of jellyfish, bovine, and fish gelatin. Understanding gelatin gel properties caused by protein or peptide content might be helpful in improving gelatin functionality in both food and non-food applications.

## Conclusions

The gel strength, viscosity, and gelling and melting temperatures of jellyfish gelatin were influenced by the concentration of HCl pretreatment and extraction time. Jellyfish which was pre-treated with 0.1 M HCl and extracted at 60°C for 12 h delivered the highest gelatin gel strength. Compared to commercial fish and bovine gelatin, jellyfish gelatin had the lowest gel strength, viscosity, and gelling and melting temperatures. The inferior gel properties of jellyfish gelatin gel might be due to a lack of 3 collagens, including collagen alpha-2 (XI chain), collagen alpha-2 (I chain), and collagen alpha-2 (IV chain), and low levels of the other 29 proteins of which 10 types of collagen and 19 non-collagen proteins.

## Supporting information

S1 Table Sequence of collagen protein in fish and bovine gelatin. (PDF)
