# Supplementary material for: Influence of collagen and some proteins on gel properties of jellyfish gelatin
Source: PLoS One. 2021 Jun 18;16(6):e0253254. doi: 10.1371/journal.pone.0253254 (PMC8216106; doi:10.1371/journal.pone.0253254)
Supplement: S1 Table — (PDF) [file pone.0253254.s001.pdf]

S 1 Table Sequence proteins in jellyfish, fish and bovine gelatin

Protein names	Organism	Sequence	Bovine	Jellyfish	JellyFish
72 kDa gelatinase (Matrix metalloproteir	Bos taurus (Bovine)	AGLLCGLVCLR	22.1093	19.2739	15.5299
Adiponectin (30 kDa adipocyte complem	Bos taurus (Bovine)	ADKNYLSK	21.2874	20.7056	19.3062
Alpha-2-antiplasmin	Homo sapiens (Humar	AARLHTQEPTPR	22.1833	19.643	19.5391
Alpha-2-HS-glycoprotein	Mus musculus (Mouse	ACFGHKQEALLNK	23.4962	20.82	19.1245
Binder of sperm protein homolog 1	Homo sapiens (Humar	AKSICDYYTGK	24.1763	20.0917	19.1194
Binder of sperm protein homolog 2	Mus musculus (Mouse	CIFPFQFK	21.2905	20.5409	20.0802
Chondroadherin	Bos taurus (Bovine)	AFSNTALVPFNR	22.5655	19.6504	19.7178
Collagen 1 alpha 2 chain	Bos taurus (Bovine)	GERGLPGVAGSV	22.4202	14.3935	13.2222
Collagen alpha 2(IX) chain	Bos taurus (Bovine)	AKREALGATGMMGPPGPPGPPGYPGK	18.0902	17.8491	12.805
Collagen alpha-2(XI) chain	Bos taurus (Bovine)	AGADGAR	0	13.8208	0
Collagen alpha-1(VIII) chain	Homo sapiens (Humar	DGLAMGK	22.5107	21.3455	18.8516
Collagen alpha-1(XI) chain	Bos taurus (Bovine)	AGPTGDPGPPGQAGEK	23.6503	22.9635	21.0623
Collagen alpha-2(I) chain	Bos taurus (Bovine)	AGPPGPPRGAGAPGQSFLLR	11.4795	16.0379	0
Collagen alpha-2(IV) chain	Bos taurus (Bovine)	AEQGEFYLLSYGSWKLNMGVPCMPEQ	16.9558	15.3143	0
Collagen alpha-2(IX) chain	Bos taurus (Bovine)	AGEKGDVGSQGVRGPQGITGPK	23.1568	22.7141	21.5673
Collagen alpha-4(IV) chain	Bos taurus (Bovine)	AAPFLECQGRQGTCHFFANK	22.03	20.8533	20.433
Collagen type IV alpha 4 chain	Bos taurus (Bovine)	AEQGELYLSYGTWR	16.5767	18.3501	14.6607
Collagen type V alpha 2 chain	Bos taurus (Bovine)	AFLTPVTKRCLR	21.0004	19.2768	17.3951
Collagen type VI alpha 2 chain	Bos taurus (Bovine)	AAMARLDK	23.462	22.4928	20.7507
Collagen type VIII alpha 2 chain	Bos taurus (Bovine)	AAASGGAAGAAGYPPAQYVQPMHK	22.3007	22.0887	19.866
Collagenase ColG	Hathewaya histolytica	AASLTNTSVTAEK	24.3342	23.5884	22.1914
High affinity immunoglobulin epsilon rec	Bos taurus (Bovine)	AATASEK	20.6647	20.07	18.4412
Integrin alpha-2	Bos taurus (Bovine)	EEGRVYLFTITKGILNWHQFLEGPNGLE	18.6469	17.0757	16.8618
Integrin beta	Bos taurus (Bovine)	ACQVCKEHEACVGLCTQCMECTPGQK	24.4959	23.5231	21.8403
Integrin beta-1 (Fibronectin receptor sub	Bos taurus (Bovine)	CNEDAQGFVCCVSSILGIWFILQLNM	19.467	18.1051	17.2722
Integrin subunit alpha 10	Bos taurus (Bovine)	AAATTWEDLSATR	24.0478	21.7027	21.7802
Matrix metalloproteinase-9	Bos taurus (Bovine)	AAFADDIVR	25.3618	22.2238	21.1822
Plasminogen	Homo sapiens (Humar	ACTFEEDEEK	24.0972	23.3253	22.0798
Prolyl endopeptidase FAP	Bos taurus (Bovine)	AAIQASNQFHVNDDATGHILLYDVR	25.1486	22.5375	20.8881
Seminal plasma protein BSP-30 kDa	Bos taurus (Bovine)	ANDLNAVFEGPACAFPFTYK	21.4264	22.1079	20.6729
Thrombin-like enzyme flavoxobin	Protobothrops flavovi	DDLVEDK	21.7179	21.1734	19.4491
Zinc metalloproteinase	Legionella pneumophi	AADPIPLQKSSFSEVTQK	22.6914	21.5507	19.6804
